# Survival analyses correlate stanniocalcin 2 overexpression to poor prognosis of nasopharyngeal carcinomas

**DOI:** 10.1186/1756-9966-33-26

**Published:** 2014-03-08

**Authors:** Shaojun Lin, Qiaojuan Guo, Jiangmei Wen, Chao Li, Jin Lin, Xiaofei Cui, Nianli Sang, Jianji Pan

**Affiliations:** 1 Department of Radiation Oncology, Fujian Provincial Cancer Hospital, Fuzhou, Fujian, China; 2 Provincial Clinical College, Fujian Medical University, Fuzhou, Fujian, China; 3 Fujian Provincial Key Laboratory of Translational Cancer Medicine, Fuzhou, Fujian, China; 4 Department of Pathology, Fujian Provincial Cancer Hospital, Fuzhou, Fujian, China; 5 Department of Biology College of Arts & Sciences, Drexel University, Papadakis Integrated Sciences Building, room 417, Philadelphia, USA; 6 Department of Pathology & Laboratory Medicine, College of Medicine, Drexel University, Philadelphia, USA

**Keywords:** Nasopharyngeal carcinoma, Radiation therapy, Stanniocalcin 2 (STC2), Biomarker, Prognosis, Retrospective, Metastasis

## Abstract

**Background:**

Stanniocalcin 2 (STC2) is overexpressed in several types of human cancers, and its overexpression positively correlates to tumor progression and poor prognosis. However, the clinical significance of STC2 overexpression in nasopharyngeal carcinomas (NPC) has not been investigated. This study examined STC2 expression in a cohort of 94 NPC samples, and explored its value in clinical diagnosis and prognosis.

**Methods:**

Tumor samples from 94 patients diagnosed in 2008 were studied. All samples were obtained prior to treatment start. All cases were clinically diagnosed and pathologically confirmed to be poorly differentiated or undifferentiated NPC without distant metastasis, and have been treated with radical radiation therapy and followed-up for five years. Survival analyses were performed.

**Results:**

Of the 94 NPC samples, STC2 overexpression (STC2+) was detected in 65 samples (69.1%). Overall survival rate of STC2 (+) patients is significantly lower than that of patients with normal STC2 levels (72.2% vs. 96.4%, respectively, *P =* 0.049). Moreover, STC2 (+) is also strongly predictive of a low progression-free survival and distant metastasis-free survival (63.0% vs 92.9%. *P =* 0.007; and 77.0% vs 96.4%. *P =* 0.028). Of the 54 patients treated with IMRT, residual tumors were found in 54.8% of STC2 positive patients (17/31), but only in 17.4% of STC2 negative ones (4/23), suggesting STC2 overexpression predicts a higher risk of residual tumors after IMRT.

**Conclusions:**

STC2 overexpression correlates to poor prognosis for NPC and may be useful as a novel biomarker to predict NPC responses to radiation. Whether STC2 promotes NPC progression and metastasis remains to be investigated.

## Background

Nasopharyngeal carcinoma (NPC) is the most commonly diagnosed head and neck malignancy in Southeast Asia. The standard treatment for NPC is radiotherapy alone for early (T1N0) disease, and combined radiotherapy and chemotherapy for more advanced lesions, including those with nodal involvement or T2–4 disease [1,2]. Recent results from conventional radiotherapy reported an overall survival rate (OSR) and locoregional control rate (LRCR) of 60-67% and 58-75%, respectively [3-7]. The adoption of intensity-modulated radiotherapy (IMRT) and combined chemoradiotherapy (CRT) has substantially improved the clinical outcomes. Results from prospective and retrospective studies have confirmed the efficacy of IMRT, and the reported LRCR of all T- and N-cases combined exceed 90%; distant metastasis becomes the main cause of treatment failure [8]. In addition, how to improve the quality of life by avoiding unnecessary dose of radiation also becomes an imminent question. Therefore, predicting of the risk of distant metastasis and patients’ response to treatment is important for the improvement of clinical management of NPC [9]. However, currently no reliable prognostic marker is available to predict treatment response and guide treatment.

Solid tumors frequently develop a microenvironment characterized by hypoxia and low glucose and glutamine supply, which contributes to gene expression reprogramming. Encoding a member of the stanniocalcin secreted glycoprotein family, STC2 is among the most upregulated genes in response to glutamine or glucose deprivation [10], and it also has been found to be upregulated under hypoxia, endoplasmic reticulum stress and radiation [11-15]. It has been proposed that STC2 overexpression (STC2+) contributes to tumor cell’s adaptation to such stress conditions, thus facilitating tumor progression [10].

It has been reported that STC2 expression was upregulated in various tumors, including breast cancer [16,17], prostate cancer [18], esophageal squamous cell carcinoma (ESC) [19], gastric cancer [20], colorectal cancer [21], renal cell carcinoma (RCC) [22] and neuroblastoma [23]. Clinical and pathological studies reveal that STC2 overexpression correlates to advanced tumor grade, tumor invasiveness, metastasis and poor prognosis in prostate cancer, ESC, gastric cancer, colorectal cancer and RCC [18]. Recently, it has also been reported that upregulated STC1, the homologue of STC2, correlates to poor prognosis in other tumors [24]. However, the expression status of STC2 and its clinical significance in NPC have not yet been investigated. We examined the STC2 expression levels in a cohort of 94 NPC samples obtained from patients prior to treatment, and carried out a retrospective study to evaluate the value of STC2 overexpression as a novel biomarker of NPC prognosis and response to radiation therapy.

## Materials and methods

### NPC samples and patients

The studied cohort includes 94 paraffin-embedded NPC samples from patients diagnosed between January and December in 2008 in Fujian Provincial Cancer Hospital, China. All cases were clinically diagnosed and confirmed pathologically as poorly differentiated or undifferentiated primary NPC without distant metastasis at the time of diagnosis. Pretreatment evaluation consisted of a complete history and physical examination, flexible fiberoptic endoscopic examination, complete blood counts, blood chemistries, urinalysis, chest X-ray, electrocardiogram, magnetic resonance imaging (MRI) scans of the head and neck, bone emission computed tomography (ECT) scans, ultrasound of liver and abdominal lymph nodes, and dental evaluation. Positron emission tomography (PET) scans, CT scans of the chest and abdomen were performed when clinically recommended.

All procedures were conducted in accordance with the Helsinki declaration, and with approval from the Ethics Committee of Fujian Provincial Cancer Hospital. Written informed consent was obtained from all participants. All cases with a Karnofsky performance status score of more than 70 were given informed consent prior to study entry. All included cases were reclassified according to the 2010 American Joint Committee on Cancer (AJCC, 7th edition). Tumor samples are obtained from 68 male and 26 female patients, with a median age of 49 year old (range 12–79). All tumor samples were obtained by biopsy prior to radiotherapy or chemoradiation therapy.

### Immunohistochemical staining

Immunohistochemical staining was carried out to determine the expression levels of STC2 protein. Sections (3 ~ 4 μm) were obtained from formalin-fixed, paraffin-embedded NPC samples from archive and mounted on pathological slides. Routinely deparaffinized in xylene and rehydrated in decreasing concentrations of ethanol to water. Antigen retrieval was achieved by boiling samples at 100°C in citrate buffer solution (Maixin_bio MVS-0101) (pH 6.0) for 10 min. The endogenous peroxidase activity was blocked by incubating sections with blocking agent (Maixin, KIT-9709/9719) for 10 min. Each slide was incubated with normal rabbit serum for 10 min at room temperature. The slides were incubated with goat anti-human STC2 antibody (1:30, R&D Systems, Inc., Minneapolis, MN 55413, USA). After washing with PBS, sections were incubated with biotin-labeled rabbit anti-goat secondary antibody at room temperature for 10 min followed by incubating with streptavidin-peroxidase for 10 min. Diaminobenzidine was used as the final chromogen, and hematoxylin was used for counter-staining.

### Radiotherapy and other treatment

Two-dimensional conventional radiation therapy (2D-RT) was utilized for 40 patients. Patients were simulated and treated supinely with a customized head shell, in 1.8 or 2 Gy daily fractions, five fractions per week for 7–8 weeks. Phase 1 consisted of two large parallel-opposed lateral faciocervical fields treating to 36–40 Gy. The fields were then taken off-cord to 50 Gy, with bilateral posterior neck matching nasopharynx dose to a total of 68–72 Gy using an anterior and two lateral fields. Node negative patients received 50–54 Gy to the neck, whereas the doses for node positive patients were boosted to 64–70 Gy at the involved nodal region. The cumulative doses to the primary lesions ranged from 60 to 80 Gy.

The other 54 patients were treated primarily with IMRT according to an IRB-approved institutional treatment protocol [25]. The gross tumor volumes (GTVs) of primary and nodal masses were obtained by CT and/or MRI. The high-risk clinical tumor volume (CTV) included the GTV plus 5–10 mm margin, encompassing the entire nasophryngeal mucosa plus 5 mm sub-mucosal volume. CTV for potentially involved regions included the posterior part of nasal cavity and maxillary sinuses, pterygopalatine fossae, posterior ethmoid sinus, parapharyngeal space, skull base, anterior third of the clivus and cervical vertebra, inferior sphenoid sinus and cavernous sinuses and the retropharyngeal lymph nodal and regions from the base of skull to the cranial edge of the second cervical vertebra. CTV of the neck nodal regions were outlined according to the recommendations by the consensus CTV delineation protocol for head and neck malignancies. An additional 3-mm margin was added to create the planning tumor volume (PTV). Brainstem, spinal cord, parotid glands, optic nerves, chiasm, lens, globes, temporal lobes, temporomandibular joints, mandible and pituitary gland were contoured and set as organs at risk during optimization. Cumulative doses of the primary lesion ranged from 69.2 to 75.25 Gy.

Ten patients out of 40 treated with 2D-RT and 22 out of 54 treated with IMRT had residual tumors after the completion of external beam radiation. A boost of 6–10 Gy was delivered using intracavitory brachytherapy, conventionally planned external beam radiation or IMRT to achieve tumor free status. Finally, a total of 71 patients received neoadjuvant, concurrent or adjuvant chemotherapy.

### Patient follow-up

All cases involved in this study have been followed up with established protocol till now or decease. Briefly, after the completion of therapy, patients were followed up at 3-month intervals during the first 2 years, at 6-month intervals from year 2 though year 5, and annually thereafter or till deceased. Each follow-up includes flexible fiber optic endoscopy, basic serum chemistry, chest X-ray and ultrasound of liver and abdomen. Either CT or MRI of the head and neck was performed after the completion of treatment and then every 6 months. The median follow-up time was 51.9 months (range, 2.1-65.6 months).

### Statistical analyses

Statistical analyses were carried out by using the SPSS statistical software package, version 18.0 (SPSS, Inc, Chicago, IL, USA). Comparisons of the distributions of clinical stages and socio-demographic of different STC2 expression groups were performed using chi-square test. Overall survival (OS), progression-free survival (PFS), locoregional relapse-free survival (LRRFS), distant metastasis-free survival (DMFS) were plotted with the Kaplan-Meier method. Multivariate analysis using the Cox semiparametric method (proportional hazard model) was performed. *P* < 0.05 was considered statistically significant.

## Results

### Establishing the standard procedures and criteria of STC2 overexpression

**Figure 1:**
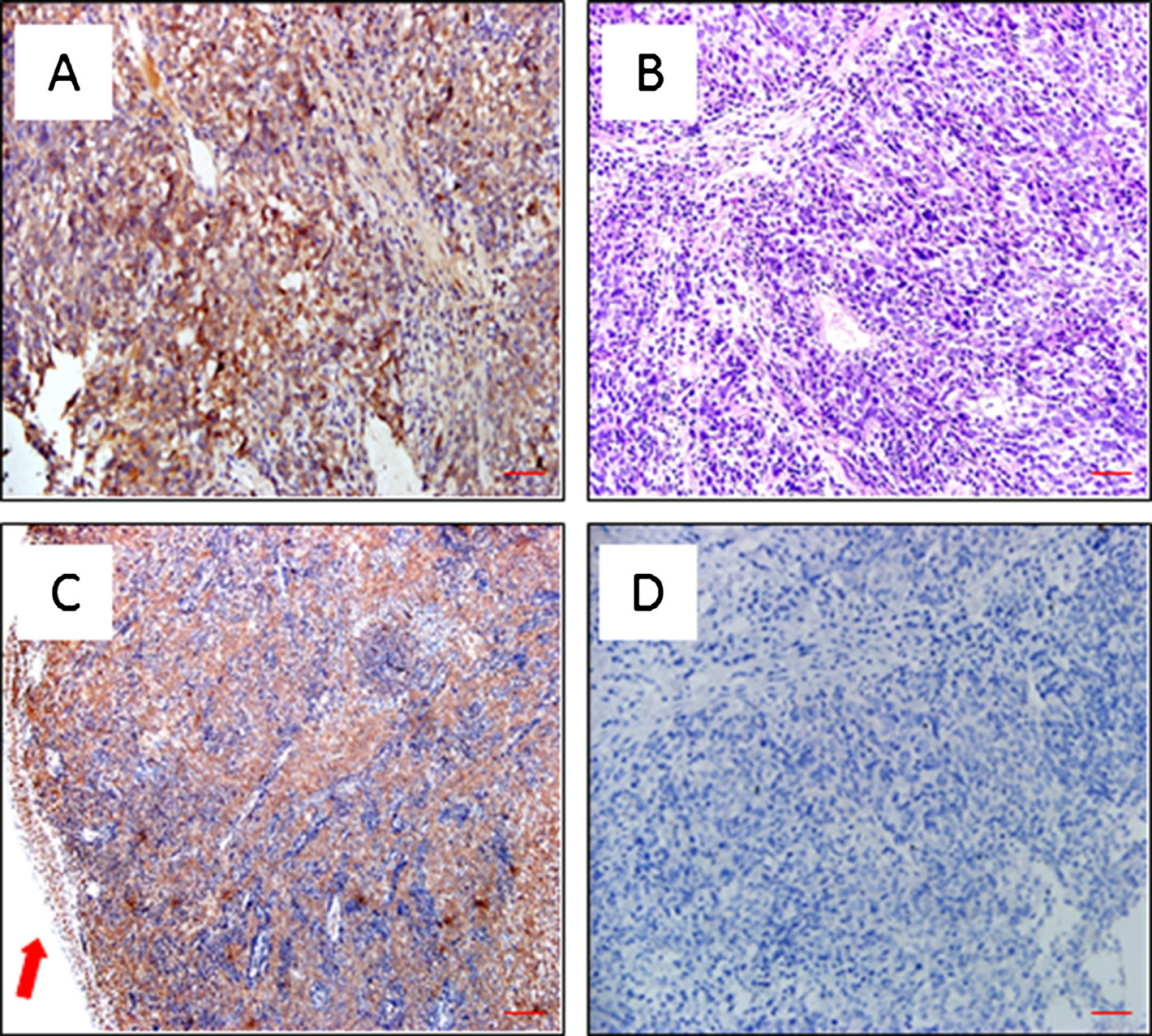
**STC2 expression in tumor adjacent nasopharyngeal epithelia with normal morphology and carcinoma cells as determined by immunohistochemistry studies. A**: STC2 expressed in local tumor (× 200). **B**: Traditional HE staining, NPC, (× 200). **C**: STC2 was overexpressed in the tumor zone (× 100). **D**: Negative Control: NPC tissue stained at the same conditions except that no specific antibody (anti-STC2) was added (× 100). Scale bar: 50 μm in **A** and **B**; 100 μm in **C** and **D**.

**Figure 2:**
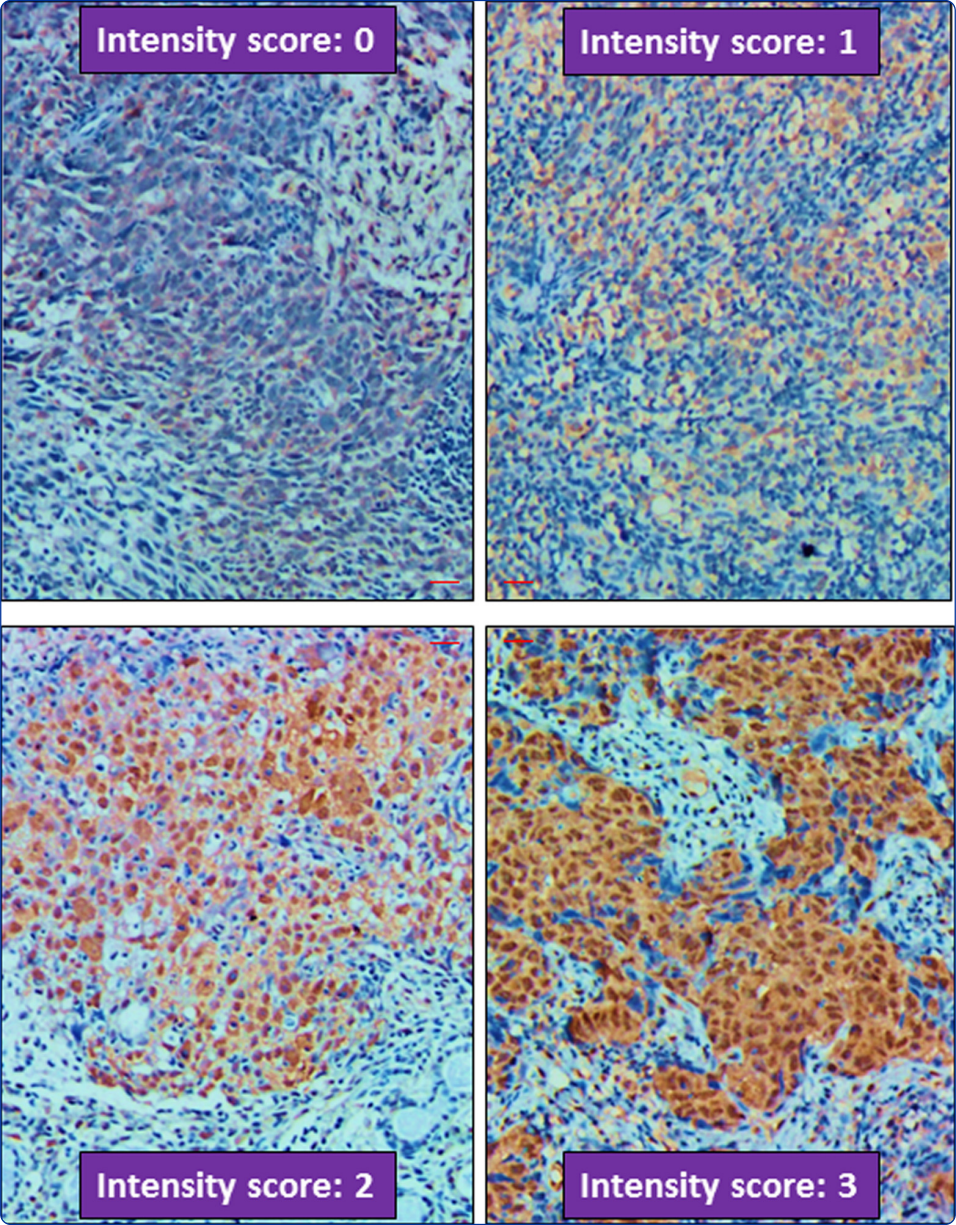
**Criteria for STC2 expression intensity scoring.** Representative micrographs were shown as labelled (× 200). All micrographs were taken and processed at identical conditions. Scale bar: 50 μm.

To establish the criteria and standards to score STC2 expression, we first stained randomly selected NPC slides to optimize the staining conditions and procedures. From this pilot study, we noticed that some tumor adjacent nasopharyngeal epithelia with normal morphology may have basal levels of STC2 expression (Figure 1). After setting the standard staining protocol, we stained all slides under the same condition, and used normal epithelia on slides stained under the same condition as internal control, and the expression levels of STC2 were scored by assessing both staining intensity and percentage of positive cell population. Brown granular staining in cells with higher intensity than normal epithelial cells was considered as positive staining (overexpression). Considering the heterogeneity of tumor tissues, the immunoreactive scores of STC2 staining were calculated as reported previously [26]. Briefly, we scored the staining intensity as: no staining (score 0), weak (score 1), moderate (score 2) and strong (score 3) (Figure 2). Percentage of positive cells was graded as following: 0–10% (grade 1), 10.1–50% (grade 2), 50.1–75% (grade 3) and 75.1–100% (grade 4). The product of intensity score and the percentage grade was used to represent the overall positivity index. Finally, we experientially defined overall positivity index 0–3 as negative and ≥4 as positive for STC2 overexpression at protein levels.

### STC2 overexpression is associated with NPC

Using this set of criteria and standards, we first addressed if STC2 overexpression was associated with NPC. Immunohistochemically stained sections were scored independently by two pathologists blinded to the clinical parameters. Among all primary tumor samples, 56 samples had both tumor and normal epithelial tissues on the same slides. The STC2 expression levels in both tumors and adjacent normal nasopharyngeal epithelial tissues were scored and positive index was calculated. Of these 56 tumor samples, the positive rate of STC2 in tumor tissues is 75.0% (42/56), and that in corresponding adjoining normal nasopharyngeal epithelial is 46.4% (26/56) (χ2 = 9.583, *P* = 0.002). When the total 94 NPC samples were taken into consideration, 65 were found to be STC2 positive, giving a positive rate of 69.1% (Table 1). These observations suggest that STC2 overexpression is associated with NPC, but is not exclusive to tumor cells.

**Table 1:** STC2 positivity in NPC and adjacent nasopharyngeal epithelia

**Groups**	**STC2 positive**	**STC2 negative**	**χ**^**2**^	***P***
Tumor cells	42 (75.0%)	14 (25%)	9.583	0.002
Adjacent epithelia	26 (46.4%)	30 (53.6%)		

### Correlation between STC2 overexpression and clinical parameters

To understand if STC2 expression levels in NPC correlate to some common clinical parameters, we grouped the samples based on different clinical parameters. We found that there was no significant correlation of STC2 positivity to gender, age, N classification, or clinical stages (Table 2). However, STC2 positive rate was higher in T3-4 than T1-2 with a marginal significance (*P*=0.050), indicating STC2 overexpression may be positively associated with increased tumor sizes.

**Table 2:** Correlation between STC2 positivity and clinical parameters of NPC

** Parameters**	**Category**	**STC2**		**χ**^**2**^	***P***
		**Positive (n = 65)**	**Negative (n = 29)**		
Gender	Male	48	20	0.239	0.625
	Female	17	9		
Age	≤50 y	37	13	1.178	0.278
	>50 y	28	16		
Histology	WHO* II	4	1	0.002	0.966
	WHO III	61	28		
T classification	1-2	16	13	3.840	0.050
	3-4	49	16		
N classification	0-1	36	14	0.407	0.523
	2-3	29	15		
Clinical stage	I	2	2	1.281	0.734
	II	8	2		
	III	34	16		
	IV	21	9		

*WHO, World Health Organization; Age in years (y).

### Correlation between STC2 overexpression and treatment response

In the cases included in this study, the choice of initial treatment was made by physicians’ recommendation and patients’ preference. As a retrospective study, we explored whether STC2 overexpression correlated to NPC sensitivity to radiation therapy, which is the primary choice of treatment for NPC currently. Upon completion of radiation therapy, disregarding the therapeutic modals, residual tumors were found in 38.5% of STC2 positive NPC (n = 65), but only 24.1% in STC2 negative cases (n = 29) (Additional file 1: Table S1). However, based on current sample size, this difference is not statistically significant. Considering the samples from patients treated with 2D-RT had an STC2 overexpression rate much higher than that from patients treated with IMRT (*P* = 0.004; Additional file 1: Table S1), we further analyzed the correlation between STC2 status and the presence of residual tumors after completion of IMRT. Of the 54 patients who received IMRT, residual tumors were found in 54.8% of STC2 positive patients (17/31), while 17.4% of STC2 negative ones (4/23). Statistical analysis indicates a significant difference (*P* = 0.014; Table 3), suggesting STC2 positivity predicts a higher rate of residual tumors after IMRT.

**Table 3:** Correlation between STC2 positivity and radiation sensitivity

**Parameters**	**Residual**	**Total**	**STC2**		**χ**^**2**^	***P***
			**Positive (n = 65)**	**Negative (n = 29)**		
IMRT	No	32	14	18	5.992	0.014*
	Yes	22	17	5		
2D-RT	No	30	26	4	0.000	1.000
	Yes	10	8	2		

*IMRT*, Intensity-Modulated Radiation Therapy; *2D-RT*, Two-Dimensional conventional Radiation Therapy; *Residual*, Presence of residual tumors. *P* value showing statistically significance was indicated by *.

### Correlation between STC2 overexpression and five-year outcome of NPC

**Figure 3:**
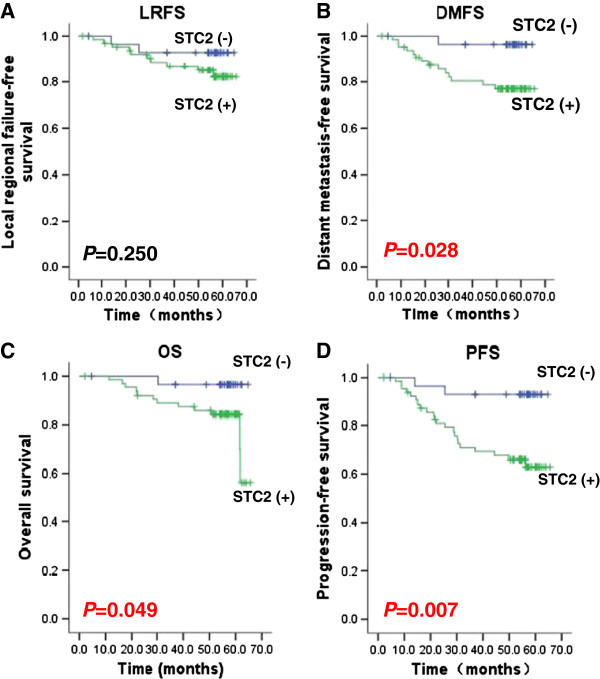
**High STC2 expression predicts inferior outcomes in NPC patients.** Survival data were analyzed and plotted using the Kaplan–Meier method. Patients were classified into STC2 negative or positive according to STC2 immunohistochemistry. *: *P* <0.05. **A**. Local regional failure-free survival (LRFS); **B**. Distant metastasis-free survival (DMFS); **C**. Overall Survival (OS); and **D**. Progression-free survival (PFS).

Four metrics are currently used clinically to describe the outcomes of NPC: overall survival (OS), progression-free survival (PFS), locoregional free survival (LRRFS) and distant metastasis-free survival (DMFS). The 5-year OS rate of the whole cohort was 80.3%, and PFS, LRRFS and DMFS were 72.4%, 85.7% and 83.0%, respectively. When compared with STC2 (+) group, the STC2 negative group had significantly higher OS, PFS and DMFS; while LRRFS showed no statistically significant difference (Figure 3, and Table 4).

**Table 4:** Correlation between STC2 positivity and 5-year outcomes

**Rate (%)**	**STC2 positive (n = 65)**	**STC2 negative (n = 29)**	**χ**^**2**^	***P***
OS	72.2	96.4	3.867	0.049*
PFS	63.0	92.9	7.236	0.007*
LRRFS	82.2	92.9	1.326	0.250
DMFS	77.0	96.4	4.854	0.028*

*OS*, Overall survival; *PFS*, Progression-free survival; *LRRFS*, Locoregional relapse-free survival; *DMFS*, Distant metastasis-free survival. *P* values showing statistically significance were indicated by *.

### Comparative analyses of STC2 overexpression and other parameters in NPC prognosis

Potential prognostic factors, including gender, age, T classification, N classification, clinical stage, the use of chemotherapy, radiotherapy modality, presence of residual tumor and STC2 status were analyzed by using the logrank test, as shown in Table 5, where numbers of events indicate failed cases regarding established censoring criteria. Statistical analyses show that neither T classification nor N classification has prognostic value for OS, PFS, LRRFS and DMFS. On the other hand, clinical stage was found to be a potential predictor for OS (P = 0.004), but not for PFS, LRRFS and DMFS. Importantly, patients in STC2 negative group showed higher OS, PFS and DMFS than STC2 positive group (*P* = 0.049, 0.007 & 0.028, respectively), suggesting STC2 overexpression is positively correlated to NPC progression and distant metastasis.

**Table 5:** Univariate logrank analyses of prognostic parameters of NPC

**Parameters**	**Category**	**No. of cases**	**OS**		**PFS**		**LRRFS**		**DMFS**	
			**Events**	***P***	**Events**	***P***	**Events**	***P***	**Events**	***P***
STC2	Positive	65	12	0.049*	22	0.007*	10	0.250	14	0.028*
	Negative	29	1		2		2		1	
Gender	Male	68	11	0.190	20	0.162	10	0.336	13	0.190
	Female	26	2		4		2		2	
Age	≤ 50 years	50	5	0.288	11	0.371	8	0.365	5	0.083
	>50 years	44	8		13		4		10	
T classification	1-2	29	2	0.151	6	0.441	5	0.411	3	0.269
	3-4	65	11		18		7		12	
N classification	0-1	50	4	0.070	11	0.270	6	0.709	6	0.200
	2-3	44	9		13		6		9	
Clinical stage	I	4	0	0.004*	0	0.472	0	0.781	0	0.317
	II	10	0		2		2		0	
	III	50	4		13		6		9	
	IV	30	9		9		4		6	
Treatment	RT alone	18	4	0.360	5	0.783	1	0.324	4	0.373
	CRT	76	9		19		11		11	
Radiotherapy	IMRT	54	3	0.008*	10	0.050*	2	0.001*	5	0.567
	2D-RT	40	10		14		10		14	
Residual	Yes	32	4	0.875	4	0.048*	2	0.194	3	0.211
	No	62	9		20		10		12	

*P values showing statistically significance were indicated by *.

“Events” are numbers of failed cases regarding OS, PFS, LRRFS and DMFS.

Univariate analyses also indicated that the addition of chemotherapy did not benefit NPC patients in any of the ending events censored. For the radiotherapy modality, IMRT may improve the treatment outcomes in regarding to OS, PFS and LRRFS (*P* = 0.008, 0.050, and 0.001, respectively), but does not significantly reduce DMFS (Table 5). Finally, patients with residual tumors at the completion of initial radiation therapy were given additional radiation (see material and methods for detail). The outcomes of this group of patients were not significantly different regarding to OS, LRRFS and DMFS, but inclined to have poor outcomes in terms of PFS (*P* = 0.048).

To more accurately analyze the covariates and to avoid the interference among variates included in this study, we next carried out multivariate survival analyses. As summarized in Table 6, T classification was found to be one of the most significant prognostic factors for LRRFS (*P* = 0.044), whereas N classification may affect PFS and DMFS (*P* = 0.044 & 0.028 for PFS & DMFS, respectively). Clinical stage seemed to be an independent prognostic factor for both OS and DMFS (*P* = 0.002 for OS and *P* = 0.037 for DMFS) (Table 6). Our Cox model also shows that STC2 status predicts unfavorable outcome regarding PFS and DMFS (*P* = 0.006 for PFS and *P* = 0.044 for DMFS, respectively), clear trends of a better OS of STC-2 negative group was also noted (*P* = 0.059, Table 6).

**Table 6:** Multivariate survival analyses of prognostic parameters of NPC

**Parameters**	**OS**			**PFS**			**LRRFS**			**DMFS**		
	**HR**	**95% CI**	***P***	**HR**	**95% CI**	***P***	**HR**	**95% CI**	***P***	**HR**	**95% CI**	***P***
STC2	7.534	0.928-61.197	0.059	7.853	1.825-33.796	0.006*	2.080	0.347-12.484	0.423	8.065	1.056-61.572	0.044*
T classification	0.610	0.105-3.534	0.582	0.590	0.180-1.932	0.383	0.176	0.032-0.954	0.044*	0.816	0.179-3.729	0.794
N classification	3.723	0.852-16.270	0.081	2.316	1.023-5.244	0.044*	1.229	0.224-6.754	0.812	4.105	1.168-14.431	0.028*
Clinical stages	6.517	2.015-21.082	0.002*	1.667	0.807-3.442	0.167	1.161	0.337-4.003	0.813	2.596	1.066-6.323	0.036*
RT vs CRT	0.278	0.051-1.507	0.138	0.906	0.268-3.067	0.874	3.561	0.286-44.327	0.324	0.364	0.081-1.632	0.187
IMRT vs 2D-RT	0.351	0.077-1.597	0.176	1.085	0.422-2.792	0.865	0.122	0.027-0.557	0.007*	5.917	1.548-22.610	0.009*
Residual tumor	0.876	0.229-3.354	0.846	0.249	0.084-0.740	0.012*	0.771	0.153-3.895	0.753	0.113	0.025-0.505	0.004*

***P***, *P* values; statistically significant ones were indicated by *; ***HR***, Hazard Ratio; **95% CI**: 95% confidence interval.

As detailed in Table 6, the retrospective data show that combined chemoradiotherapy apparently resulted in a higher DMFS than radiotherapy alone (*P* = 0.028). Finally, IMRT was found to improve LRRFS and DMFS significantly (*P* = 0.007 & 0.006 for LRRFS & DMFS, respectively).

## Discussion

The clinical outcome of NPC has been significantly improved since the introduction of IMRT and other new therapies. The major obstacle for successful treatment of NPC is that some NPC cases tend to become progressive and distant metastasis. A better understanding of the molecular mechanisms underlying NPC progression and distant metastasis may nominate new therapeutic targets. For example, a recent study focused on the role of MTA1 in NPC progression [27]. On the other hand, identifying prognostic biomarker may facilitate the prediction of response prior to treatment initiation, thus guiding the choice of treatment.

STC2 has been reported to be upregulated in a series of cancers and been correlated to tumor progression and prognosis [18]. It has been reported to be upregulated in response to various stresses, including hypoxia, endoplasmic reticulum stress, oxidative stress and radiation [11,12,28,29]. However, whether it is overexpressed in NPC has not been investigated. We attempt to evaluate the expression status of STC2 and its correlation to long-term outcomes of NPC patients. We reported that the expression level of STC2 in primary NPC cells is significantly higher than that in corresponding normal nasopharyngeal epithelia. The 5-year OS, PFS and DMFS rate of the STC2-negative group is significantly higher than that of STC2-positive group. These results are of particular importance, as they represent the first study showing the expression status of STC2 in NPC may be a potential prognostic biomarker, which may be used to identify patients with potentially unfavorable outcomes prior to the initiation of treatment; these patients may benefit from a more intensive therapeutic regimen.

It has been reported that STC2 overexpression is associated with poor prognosis or cancer recurrence in most of the cancers studied [18]. In ovarian cancers, STC2 was one of the overexpressed genes as investigated by immunohistochemistry-guided laser capture microdissection and microarray; and overexpression of STC2 was associated with a decreased disease-free interval [30]. Increased cytoplasmic STC2 expression correlated to aggressiveness of renal cell carcinoma and shorter overall patient survival times [22]. In colorectal cancer, it was reported that STC2 was more frequently overexpressed in cancerous tissues than in non-cancerous tissues, and high mRNA expression of STC2 was significantly associated with tumor sizes, depth, lymph node metastasis, lymphatic permeation, AJCC stage classification and overall survival. In addition, STC2 expression was found to be a factor affecting overall survival rate by multivariate analysis [21]. In gastric cancer, patients in the high STC2 expression group had a significantly poorer overall survival than those in the low STC2 expression group [20]. We found that high STC2 expression was significantly associated with higher T classification of NPC, and STC2 was shown to be a prognostic factor predicting poorer OS, PFS and DMFS of NPC.

However, STC2 overexpression in breast cancers, particularly in estrogen receptor-positive breast cancers, is associated with favorable prognoses [16,31-33]. Estrogen receptor-positive breast cancers are usually low-grade malignancies and can be effectively treated with hormonal therapies, which may explain the favorable prognosis [34,35].

Whether STC2 overexpression contributes to tumor progression and distant metastasis remains unknown. Tumor progression depends on accelerating the utilization, and sustaining sufficient supply, of nutrients including molecular oxygen, carbon source and nitrogen sources [36,37]. Reprogrammed carbon source utilization during tumor progression has been well known for decades; it has been established that oncogenic transformation promotes the utilization of glutamine [38,39]. However, solid tumors frequently develop a microenvironment characterized by hypoxia, low glucose and glutamine supply. As one of the most upregulated genes in response to glutamine or glucose deprivation, STC2 may participate in triggering adaptive tumor metabolism, hence contributing to tumor progression and distant metastasis [10]. However, the precise role of STC2 in tumor progression and metastasis remains to be investigated by further studies including loss-of-function analysis. Nevertheless, our data show that STC2 overexpression correlates to NPC progression, which is consistent with previous studies in other solid tumors [18] and with the notion that STC2 overexpression facilitates tumor progression and migration.

In this study, we found that STC2 (+) NPC resulted in a higher rate of residual tumors than STC2 (−) group at the completion of initial IMRT, suggesting STC2 overexpression may contribute to tumor cell resistance to radiation therapy. However, the molecular mechanisms underlying the STC2 overexpression in NPC and radiation resistance remains to be further investigated. It has been reported [11-15] that STC2 expression is induced by oxidative stress and hypoxia, and STC2 overexpression contributes to antiapoptotic activity and survival of ischemia nerve cells. Solid tumors frequently develop a hypoxic condition which activates HIF-1 [40] to sustain tumor growth and progression and STC2 is one of HIF-1 target genes. Moreover, STC2 functions to protect cells from apoptosis in hypoxic ovarian cancer cell lines [11,12,28,29]. In addition to hypoxia, radiation represents another stress and source of oxidative stress to tumor cells, which may lead to the upregulation of STC2 following IMRT. If proven, targeting the protective activation of STC2 may be explored as a novel strategy to improve radiation sensitivity.

In addition to attenuating cell proliferation and reducing cell apoptosis, enhancing cell migration under stress conditions by STC2 has been reported. It has been proposed that STC2 facilitates tumor cell migration through epithelial-mesenchymal transition (EMT) and upregulation of MMP-2 and MMP-9 in hypoxic conditions [14,23]. Our observation that STC2 (−) NPC patients have a significant higher DMFS than STC2 (+) patients is consistent with the concept that STC2 overexpression promotes distant metastasis.

In this retrospective study, most patients received the institutional chemotherapy regimen for NPC, and choice of chemotherapy plans (i.e., concurrent, neoadjuvant, adjuvant or any combination) was at the discretion of the attending physicians. A total of 71 (75.5%) patients received neoadjuvant chemotherapy, 34 (36.2%) and 38 (40.4%) patients received concurrent chemotherapy and adjuvant chemotherapy, respectively. We note that the use of chemotherapy may influence the outcomes of treatment, hence affecting the accuracy of data interpretation. Carefully controlled prospective studies in the future may complement this study.

## Conclusions

In conclusion, we provide data to show that STC2 overexpression correlates to NPC malignancy and poor prognosis including higher potential of progression and distant metastasis. STC2 overexpression may be a novel prognostic biomarker. Its clinical value as a therapeutic target for treatment of NPC patients remains to be investigated. Future investigations including prospective studies are required to further validate the value of STC2 overexpression in NPC prognosis. Laboratory animal and cell culture studies are also required to provide mechanistic understanding of the roles of STC2 in tumor progression, metastasis and resistance to radiation therapy (IMRT).

## Abbreviations

2D-RT: 2-dimensional conventional radiation therapy; CTV: Clinical tumor volume; DMFS: Distant metastasis-free survival; ECT: Emission computed tomography; ESC: Esophageal squamous cell carcinoma; GTV: Gross tumor volumes; IMRT: Intensity-modulated radiotherapy; LRCR: Locoregional control rate; LRRFS: Local regional relapse-free survival; MRI: Magnetic resonance imaging; NPC: Nasopharyngeal carcinomas; OS: Overall survival; PFS: Progression-free survival; PET: Positron emission tomography; PTV: Planning tumor volume; RCC: Renal cell carcinoma; STC2: Stanniocalcin 2.

## Competing interests

The authors declare that they have no competing interest.

## Authors’ contributions

SL participated in the design and coordination of this study, and helped draft the manuscript. QG designed experimental protocols and drafted the manuscript. JW carried out the Immunohistochemical staining, performed the statistical analysis and participated in manuscript preparation. CL participated in the data collection and Immunohistochemical staining. JL and XC participated in the data collection. NS and JP conceived of this idea, outlined study design, and helped manuscript preparation. All authors have read and approved the final manuscript.

## Supplementary Material

Additional file 1: Table S1 STC2 overexpression status in different treatment groups. Click here for file
