# Supplementary material for: Survival analyses correlate stanniocalcin 2 overexpression to poor prognosis of nasopharyngeal carcinomas
Source: J Exp Clin Cancer Res. 2014 Mar 8;33(1):26. doi: 10.1186/1756-9966-33-26 (PMC4015363; doi:10.1186/1756-9966-33-26)
Supplement: Additional file 1: Table S1 — STC2 overexpression status in different treatment groups. [file 1756-9966-33-26-S1.pdf]

Supplementary Table
STC2 overexpression status in different treatment groups

Parameters	Category	STC2 Positive (N=65)	STC2 Negative (N=29)	χ^2	*P*
Treatment	RT alone	14	4	0.777	0.378
	CRT	51	25		
Radiotherapy	IMRT	31	23	8.201	0.004
	2D-RT	34	6		
Residual	Yes	25	7	1.832	0.176
	No	40	22		

RT: radiation therapy; CRT: chemoradiation Therapy; IMRT: intensity-modulated radiation therapy; 2D-RT: Two - dimensional radiation therapy; Residual: Presence of residual
